# “I made friends a lot more easily”: children and families’ experiences of social group programs for children on the autism spectrum

**DOI:** 10.1186/s12887-025-05686-6

**Published:** 2025-05-03

**Authors:** Maya Hayden-Evans, Bahareh Afsharnejad, Elinda Ai Lim Lee, Ben Milbourn, Tanya Picen, Mathew Johnson, Sven Bölte, Sonya Girdler

**Affiliations:** 1 Curtin School of Allied Health, Curtin University, Perth, WA Australia; 2 Curtin Autism Research Group (CARG), Curtin University, Perth, WA Australia; 3 Curtin School of Population Health, Curtin University, Perth, WA Australia; 4 Autism Association of Western Australia, Perth, WA Australia; 5 Center of Neurodevelopmental Disorders (KIND), Centre for Psychiatry Research; Division of Neuropsychiatry, Department of Women’s and Children’s Health, Karolinska Institutet & Child and Adolescent Psychiatry, Stockholm Health Care Services, Stockholm County Council, Stockholm, Sweden; 6 School of Allied Health, University of Western Australia, Crawley, WA Australia

**Keywords:** Social groups, Autism, Children, Parents, Intervention, Lived experience

## Abstract

**Background:**

Social Skills Group Programs (SSGP) target the social communication and interaction skills of children on the autism spectrum. This qualitative study explored lived experiences of children and families who participated in a randomised controlled trial (RCT) evaluating the efficacy of KONTAKT™ adapted for younger children (8 to 12 years) in comparison to an active control social art group (ART Legends).

**Methods:**

Semi-structured interviews were conducted online with parents (*n* = 37) and children (*n* = 35) who participated in the RCT, immediately following the interventions. Interview questions were designed to elicit responses relating to program content, structure, and experiences. The data were analysed using a deductive coding framework.

**Results:**

Findings suggest SSGPs such as KONTAKT™, implementing multiple teaching strategies, and less structured social group activities such as ART Legends can both have a perceived positive influence on outcomes. More children in the KONTAKT™ group reportedly improved their social skills than those in the art group. Overall, participants’ experiences were predominantly positive. Both barriers (e.g., session timing, distance from home) and facilitators (e.g., support of family members) to participation were identified; feelings towards individual aspects of the groups were dependent on a range of personal factors (e.g., existing commitments).

**Conclusion:**

This study describes experiences of organised social group participation from the perspectives of children on the spectrum and their families, supporting the positive influence of such contexts for autistic youth. Suggestions made by participants to improve social groups are presented, contributing to ongoing development of SSGPs for children on the spectrum.

**Trial registration:**

(1) Australian New Zealand Clinical Trials Registry (ANZCTR): ACTRN12619000994189, registered 12 July 2019, anzctr.org.au; (2) ClinicalTrials.gov: NCT04024111 registered 1 December 2019, https://clinicaltrials.gov.

**Supplementary Information:**

The online version contains supplementary material available at 10.1186/s12887-025-05686-6.

## Background

Challenges in social communication and social interaction skills in mainstream societal settings are among the defining features of autism, a neurodevelopmental condition [1]. These challenges can impact an individual’s functioning in everyday life, limiting participation in key areas including education, employment, and relationships [1]. Global estimates of prevalence indicate that one in 100 children have an autism diagnosis [2]. In Australia, the prevalence of autism is currently highest among school-aged children [3], highlighting a critical need for support and programs aiming to increase social participation and wellbeing that are tailored towards the unique needs of this age group.

The school-aged years are a period of critical development for building relationships, establishing a sense of identity, and facilitating academic success [4–6]. Today, autism increases the risk of school-aged children experiencing negative outcomes in relation to mental health and academic achievement, impacting their future employment opportunities [7–9]. While the neurodiversity movement [10] and research taking a functional approach to autism increasingly highlight the role of the environment in enabling the outcomes of autistic individuals [11], there is still a need for programs empowering autistic individuals to develop their own social opportunities.

Social skills group programs (SSGPs) have a long history in autism research and practice, and are among the most evidence-based approaches to developing social competencies of children on the spectrum [12, 13]. SSGPs commonly target children and adolescents on the spectrum with cognitive abilities in the average range (IQ > 70) and are typically delivered by clinicians to small groups comprising up to eight children or adolescents [14]. SSGPs for more naturalistic settings, such as school, have also been developed [15]. There is a growing body of evidence supporting the efficacy of SSGPs for young people on the spectrum in relation to improvement of social knowledge and performance outcomes [13, 16, 17]. However, there is limited data supporting the acceptability of SSGPs from the perspective of those they seek to impact, including both children on the spectrum and their caregivers. While a few studies have incorporated young people’s lived-experience of participating in SSGPs [18–20], further exploration of the factors affecting outcomes is needed to better understand the utility of these programs [21].

The efficacy of SSGP, KONTAKT™, has been evaluated via randomised controlled trials (RCT) with Swedish youth, aged 8 to 18 years [22] and Australian adolescents, aged 12 to 17 years [23]. The Swedish evaluation indicates that KONTAKT™ contributes to improving adaptive functioning and reducing the presentation of autistic traits, to varying degrees, in adolescents on the spectrum when compared to treatment as usual, depending on dosage and participants’ characteristics [22]. Similarly, an Australian study of the program found that KONTAKT™ participants showed greater social improvements than their autistic peers who attended a less explicit comparative social group [23]. Generally, findings of RCTs evaluating the efficacy of KONTAKT™ with younger children (aged 8 to 12 years) in both Sweden and Australia have been less conclusive [22], (Afsharnejad B, Lee EAL, Hayden-Evans M, Fridell A, Coco C, Johnson M, et al: Which should I attend: KONTAKT Social Skills Group Program or ART Legends Social Group? A double-blinded randomised controlled trial, in preparation). Given this lack of clarity, research exploring the experiences of younger KONTAKT™ participants and their parents is needed to inform further tailoring of the program towards the needs of autistic children. While a qualitative study has investigated the experiences of a small sample of Swedish children on the spectrum and their caregivers who participated in KONTAKT™, a year after the program [19], to date only the experiences of Australian adolescents and their caregivers have been qualitatively investigated immediately following the intervention [18].

While program evaluation is primarily driven by quantifiable changes in participants’ performance on outcome measures, qualitative methods of investigation have an important role in highlighting the meaning and experiences associated with these changes [24]. Although it can be helpful to know that autistic individuals who participate in social skills programs are likely to experience an increase in social knowledge, skills and goal attainment, it is equally as important to understand the process, including why and how this occurs from the perspective of participants, to better understand the essential components of a program, and continue to develop and improve interventions for the populations they are designed to benefit [24]. The SSGPs included in this study utilised a variety of teaching strategies to foster social skills practice amongst children on the spectrum, including both explicit (e.g., didactic) and implicit (e.g., eliciting social behaviour) strategies [25]. Both groups involved a combination of both approaches, however, KONTAKT™ is designed to include more explicit social skill instruction through themed discussions and role play activities, while the control group, ART Legends, employed a primarily implicit approach, intending to elicit social behaviour through shared interaction and participation in a social group activity, with minimal explicit social skill instruction.

Therefore, the aim of this study was to explore the lived experiences of Australian children and families who participated in a RCT evaluating the efficacy of the 16-session variant of a blended approach SSGP, KONTAKT™, adapted for younger children, in comparison to a more implicit social control group, ART Legends. This included exploring participants’ experiences of the two different types of programs, their satisfaction with the program they participated in, and the reported outcomes observed by both caregivers and the children themselves.

## Methods

### Design

This qualitative, exploratory study drew participants from a multisite, pragmatic, double-masked, parallel group RCT evaluating the effectiveness of a 16-session variant of KONTAKT™ in comparison to an active control group (ART Legends) for children aged 8 to 12 years in Australia  (Afsharnejad B, Lee EAL, Hayden-Evans M, Fridell A, Coco C, Johnson M, et al: Which should I attend: KONTAKT Social Skills Group Program or ART Legends Social Group? A double-blinded randomised controlled trial, in preparation). The RCT was designed to adhere to the Consolidated Standards of Reporting Trials (CONSORT) guidelines [26]; the methods are reported in full elsewhere (Afsharnejad B, Lee EAL, Hayden-Evans M, Fridell A, Coco C, Johnson M, et al: Which should I attend: KONTAKT Social Skills Group Program or ART Legends Social Group? A double-blinded randomised controlled trial, in preparation). As with the Australian RCT evaluating KONTAKT™ for autistic adolescents, the primary outcome of the RCT involving children was their progress towards achieving their personally meaningful social goals, measured using Goal Attainment Scaling (GAS; [27]). Aligning with previous qualitative studies exploring participants’ experiences of KONTAKT™ [18, 19], this study utilised a primarily deductive approach [28], following the steps outlined by Assarroudi and colleagues [29]. Semi-structured interviews were conducted with participating children and their parents to determine their perspectives on the content and structure of the social group they participated in, and deductively analysed and organised into themes according to a framework informed by the findings of the previous studies [18, 19]. Inviting a larger sample, including both parents and children, to participate in this qualitative study enabled adequate exploration and contrast of the experiences of each participant type across the different intervention groups [30]. The current study was approved by the Curtin University Human Research Ethics Committee (HRE2017 - 0245).

### KONTAKT™ social skills group program

Originally developed in Germany, KONTAKT™ is a manualised SSGP underpinned by principles of cognitive behavioural therapy, behavioural activation, psychoeducation, observational learning, and parent involvement [31]. The program follows an agenda with each session of KONTAKT™ consisting of the same components, outlined in Table 1. These sessions incorporate both structured and unstructured components, employing a combination of implicit and explicit teaching strategies to assist autistic youth in practising their social skills. The program targets social interaction and communication skills, social motivation, awareness of self and others, problem-solving, and self-confidence. Initially adapted to the Swedish cultural context with short (12 sessions; [22]) and long (24 sessions; [32]) variants, KONTAKT™ has since been adapted to a 16-session variant aligned with the Australian culture [33]. A RCT has been conducted with both Australian autistic adolescents [23] and children (Afsharnejad B, Lee EAL, Hayden-Evans M, Fridell A, Coco C, Johnson M, et al: Which should I attend: KONTAKT Social Skills Group Program or ART Legends Social Group? A double-blinded randomised controlled trial, in preparation) at autism service centres. Participants in the current study attended the 16-session program which ran once per week over two school terms (8 sessions per term), for a period of 75 min each. Each session was facilitated by two trained health professionals who had backgrounds in occupational therapy, social work, or psychology, with groups comprising between five and eight children.

**Table 1:** Components of KONTAKT™ social skills group program [31]

Component	Description
Opening round	Welcome and transition into the session, promoting interaction between group members
Reviewing mission	Reviewing homework activities assigned in previous session
Themed group discussion	Encouraging exchange of relevant experiences, promoting social interaction between group members, implementing conversational skills and practising managing conflicts
Snack break	Encouraging social skills in unstructured social environments (e.g., small-talk, turn-taking)
Group activity	Facilitating development and practice of social skills (e.g., role play, emotion-processing, social games)
Assigning mission	Facilitating generalisation of skills discussed in the group to other social situations outside of the group
Closing round	Conclusion and transition out of the session, promoting interaction between group members

### ART Legends social art group program

ART Legends is a structured, purposely-designed social group with the same dosage and exposure as the intervention group, aiming to support children on the spectrum to engage in art projects in a safe social context. Facilitated by health professionals at autism service centres, this program did not explicitly teach social skills, but instead provided children with incidental opportunities to socially interact with their peers through shared engagement in a variety of art projects. The program was designed to follow a similar structure and routine each week as outlined in Table 2.

**Table 2:** ART Legends should have capital letter as this is the name of the program

Component	Description
Opening round	Welcome and transition into the session, setting up for art activity
Discussion	Discussing the art theme of the week
Arts and craft	Working on the week’s art project
Snack break	Having a snack and socialising in an unstructured setting
Arts and craft	Continuing with the week’s art project
Show and tell	Sharing the art with the group, asking questions of others
Closing round	Cleaning, packing up, and transitioning out of the group

### Participants

Autistic participants reported on in this study were recruited from the 84 families randomised to the KONTAKT™ and ART Legends groups conducted between July 2020 and June 2022 (ACTRN: 12,619,000,994,189). Participants in the RCT had 1) a diagnosis of autism in Australia according to the Diagnostic and Statistical Manual of Mental Disorders (DSM; 1), confirmed by records and via parent proxy-report on the Autism Treatment Evaluation Checklist [34]; 2) an IQ > 70 determined by the Wechsler Intelligence Scale for Children, online [35, 36]; and 3) were motivated to participate in a SSGP, assessed during screening. All eligible RCT participants and their parents were invited to be interviewed immediately following the interventions. A total of 35 participating children [KONTAKT™: *n* = 17 (48.6%); ART Legends: *n* = 18 (51.4%)] and 37 parents [KONTAKT™: *n* = 17 (45.9%); ART: *n* = 20 (54.1%)] participated in the interview, collectively representing 39 children. This included one parent with two children and one child whose parent did not participate in the interview. The data included four participants (KONTAKT™, *n* = 3; ART, *n* = 1) who withdrew from the program after attending a few sessions. The mean age of the children was 10.7 years (SD = 1.3) for the KONTAKT group compared to 10.0 years (SD = 1.2) for the ART Legends group. Nearly 80% of the participants were male for both groups [KONTAKT™, *n* = 15 (78.9%), ART Legends, *n* = 17 (85%)]. The majority of the children were diagnosed with autism spectrum disorder under the DSM- 5 [KONTAKT™:, *n* = 16 (84.2%); ART Legends: *n* = 15 (75%)]. The mean age for the KONTAKT™ parents was 47.0 years (SD = 19.4) and 41.7 years (SD = 4.9) for the ART Legends parents. Nearly all parents in both groups were mothers, except for one father in the KONTAKT™ group. Table 3 presents the demographic and clinical characteristics of the families involved in the RCT who agreed to be interviewed for this qualitative study.

**Table 3:** Demographic and clinical characteristics of participants interviewed post-intervention

Demographic and clinical characteristics	KONTAKT™	Art Legends
Children		
Number of children, n (%)	19 (48.7%)	20 (51.3%)
Age (years), M (SD)	10.7 (1.3)	10.0 (1.2)
Males, n (%)	15 (78.9%)	17 (85%)
Full-scale IQ	103.2 (11.9)	103.6 (14.7)
Autism diagnosis, n (%)		
DSM- 5	16 (84.2%)	15 (75%)
DSM-IV	3 (15.8%)	5 (25%)
^ a^Attendance percentage, M (SD)	75.4 (38.3)	90.8 (20.9)
Parents		
Number of parents, n (%)	17 (45.9%)	20 (54.1%)
Mother	17 (100%)	19 (95%)
Father	0 (0%)	1 (5%)
Age (years), M (SD)	47.0 (19.4)	41.7 (4.9)

*M* Mean, *SD* Standard Deviation, *IQ* Intelligence Quotient, *DSM- 5* Diagnostic and Statistical Manual of Mental Disorders, 5 th Edition, *DSM-IV* Diagnostic and Statistical Manual of Mental Disorders, 4 th Edition

^a^The proportion of attendance for each participant was calculated based on the 16-week program

### Attendance

The RCT was conducted during the COVID- 19 pandemic, which impacted attendance, with some children unable to attend due to being unwell, or someone in their immediate household was unwell and therefore the child was unable to attend due to government-enforced restrictions at this time. Despite this, attrition rates in the broader RCT were on par with evaluations of other SSGPs [13], as well as the earlier evaluation of KONTAKT™ in Australia [23]. The attendance rate of both groups is presented in Table 3.

### Data collection

All caregivers provided written informed consent for both themselves and their child (or children) to participate in the study prior to commencement of data collection. Immediately following the conclusion of the KONTAKT™ and ART Legends groups (post-test; 16 weeks), semi-structured interviews were conducted online via Microsoft Teams by a research team member who was not masked to group allocation. The semi-structured interview guide was developed to align with questions asked of participants in the previous qualitative exploration of KONTAKT™ for teenagers [18], broadly covering the categories of structure (e.g., number of sessions, information received, group size), content (e.g., thoughts on each round of the program, activities completed) and experiences (e.g., changes in social skills, program satisfaction, positive or negative impacts of participating). Participants’ interviews were video and/or audio recorded with their consent. A visual guide, which included the interview questions and visual scales and prompts, was shared with the children throughout the online interview to facilitate communication with them. These are included as Supplementary Materials.

### Data analysis

The demographic and clinical characteristics of participants in KONTAKT™ and ART Legends were descriptively analysed. Interviews were recorded and later transcribed using Otter.ai. The transcriptions were manually reviewed for accuracy and edited as required, including de-identifying participants, before being imported into Nvivo [37] for coding. The 16-step approach to directed content analysis described by Assarroudi and colleagues [29] was used to prepare, organise and report the findings. This is a deductive method commonly used in health-related studies to develop a framework based on previous knowledge [38]. In this study, the coding framework was derived from the Australian qualitative study by Afsharnejad and colleagues [18], which organised codes under the main categories of program-related factors, person-related factors, and factors affecting participation, guided by the International Classification of Functioning, Disability and Health (ICF) framework [39]. Initial coding was conducted by multiple authors (BA, EL, MHE, TP) in Nvivo and reviewed, combined and finalised by BA, who also organised the codes into themes aligning with the predetermined coding framework, and inductively identified any new themes unique to this study. The resulting themes were reviewed by the other members of the research team and member-checked by family members of autistic individuals.

## Results

Using the coding framework initially developed by Choque Olsson and colleagues [19] and built upon in the Australian qualitative study by Afsharnejad and colleagues [18], participants’ responses were categorised as 1) Program-related factors; 2) Person-related factors; 3) Factors affecting participation; 4) and Research-related factors.

### Program-related factors

Program-related factors encompassed participants’ perspectives of the structure and content of the SSGPs, as well as their overall satisfaction with the program they participated in. These factors were organised into the following themes: overall satisfaction, session structure, group structure, and content.

#### Overall satisfaction

Most parents described their overall experiences of the SSGPs positively (89%). Children were reportedly “happy to go” and “really enjoyed it”. However, overall satisfaction was higher among the parents of children in the ART Legends group (100%) than KONTAKT™ (78%). Satisfaction may have been further improved by an increase in information from staff about the program, greater communication with parents, and more feedback regarding the child’s participation during the sessions:

I think everyone would have appreciated just a bit more communication throughout the whole course. Like, the fact that the 16 weeks went by and we didn’t really get anything, just like, you know, one of the COVID emails saying, you know, we’re not doing it. And then now we’re doing it again, kind of thing. That’s all we got. So yeah, I think that could have helped. (Parent-KONTAKT^TM^).

Almost half (46%) of all parents cited the opportunity to interact with other children on the spectrum as helpful for their child. This was the most frequently reported helpful factor for parents of children in the ART Legends group (63%) while parents of children in KONTAKT™ reported learning and applying skills to improve their child’s confidence, was most helpful (44%). Overall satisfaction was similarly positive for the children (86%), with those in the KONTAKT™ group reporting marginally higher rates of satisfaction (89%) than those in the ART Legends group (81%). Children who participated in the KONTAKT™ group were more likely to recommend the program to other children on the spectrum (89%) than those who participated in ART Legends (50%).

I have to say I was really impressed with the program. I think it’s probably one of the best programs that [child] has ever done. I feel like, because he’s done other programs, social skills groups and activity groups which were Lego based, and they were really like, you know, just once in the holidays or twice in the holiday, and I don’t feel like it really had much to any effect. I really feel like this program had a significant positive effect for [child]. Yeah, so I would definitely recommend it, and I’d love to see it running again. (Parent-KONTAKT^TM^)

#### Session structure

Most parents (97%) commented on the overall structure of the program and sessions. These ideas were arranged under the following subthemes: number of sessions, parent information, and parent sessions.

##### Number of sessions

Approximately half of all parents (53%) were satisfied with the programs running for 16 sessions. Some parents compared the length of these SSGPs to other, similar programs they had done in the past, indicating that “normally when you do a group, it’s normally like nine sessions, sometimes ten, and the 16 was really good.” Parents suggested the longer duration of the program enabled the children to “get to know” the others in the group and provided “some level of consistency”. Some parents (26%) of children in the ART Legends group would have liked the program to continue as their child “really looked forward to it.”

##### Parent information

Although many parents (73%) indicated they received enough information before the programs started, some parents [KONTAKT™ (K): 33%; ART Legends (A): 26%] would have liked to know “more detail up front about what they’d be doing in the sessions”. Some parents (41%) also expressed that it may have been beneficial to have more feedback throughout the program.

We didn’t receive any information about progress at all. And that was something that all the mums bought up at our last parent meeting, the fact that, that it could have been good at the end of each session, just to have a quick few seconds: Oh, this happened, this happened or even like a generic email - This week, we covered this - or just something, because we had no idea…(Parent-KONTAKT^TM^)

##### Parent sessions

The KONTAKT™ program included three parent sessions, which half of the parents agreed were “enough” and provided them with an opportunity to “touch base”. Parents also mentioned that the group facilitators were generally available to talk to at the end of the session and “if there was anything important that came up, the people running the program would ask you to come in”.

#### Group structure

Almost all parents (95%) and some children (23%) discussed elements relating to the group structure and dynamics. These were organised under the subthemes of age and gender, group size, and group dynamics.

##### Age and gender

Around half of the parents (51%) were happy with the age range included in the programs (8–12 years), with more parents of children in the ART Legends group (58%) reporting satisfaction with the range than those in KONTAKT™ (44%). A number of parents of children in KONTAKT™ (33%) expressed a desire for the children in the group to be around the same age. One parent commented, “it’s a big age gap, and very different social aspects at either end of those scales.” One of the children in the KONTAKT™ group reported they were the “oldest” and “there wasn’t enough variety”, so they felt they “couldn’t relate to anyone”. There were more males than females included in the study. One child who participated in the KONTAKT™ group said she was “the only girl in the group, so that was a bit awkward.”

##### Group size

A greater proportion of parents of children in the art group (65%) reported satisfaction with the group size than those whose children were in KONTAKT™ (47%). Some parents (32%) suggested larger groups would have been more beneficial to help the children “meet more variety of people”.

I think more would have been better, when there were times that there might have been only a few kids at the session, because there were a few kids away... And I think just having more, if it’s possible, just changes the dynamic. They get more time to talk and collaborate with other kids who are, you know, totally different, on the spectrum. (Parent-KONTAKT^TM^)

However, more than half the children (51%) reported high satisfaction with the group size, saying smaller groups had “less noise” and were less “cramped”, and “everything just gets really crowded” when groups are larger. Similarly, one parent suggested that their children would prefer a smaller group, saying:

I think other people would say that there wasn’t enough. But I think that my kids do better in smaller numbers and are very well taken care of, and I think the staff got to know them very, very well. So, I don’t know that I would necessarily want too much, too many more. (Parent-KONTAKT^TM^)

##### Group dynamics

Both parents and children commented on the dynamics of the group, from their perspectives. Children generally described the other members of their group as “nice” and considered them “friends”. However, some children in the group could be “annoying” and “loud”. Parents based their opinions of the group dynamics on observation (e.g., “…in the parent one, when we sat and watched, there was lots of silliness) and information from their children.

He does feel quite a sense of belonging, he says, “I like being with other autistic people because I feel comfortable, like we all sort of understand each other. We all understand that we’re a bit different and we don’t get upset at each other for that.” (Parent-ART Legends)

Other parents also emphasised the importance of their child connecting with others on the spectrum, saying “they all felt comfortable with each other, with their little quirks and differences, and were comfortable just being themselves…it was a really nice experience for him to not feel quite so different.”

#### Content

Participants’ thoughts on the content of the SSGPs was organised according to the overall structure of the sessions. The art program followed a similar structure to KONTAKT™, which adheres to a specific structure each session comprising an opening round, activity, discussion, homework, snack time, and closing round. Unique to the different programs, ART Legends participants reflected on the show and tell component and on packing up and cleaning away art supplies, while the KONTAKT™ participants shared their experiences of the excursion and moderating a session. Parents perceptions of the content of the programs was limited as they were not present for the majority of the sessions and instead based their opinions on information provided by their children, the group facilitators, and the information they received about the programs.

##### Opening/closing round

Children were mostly satisfied with the opening/closing round, indicated by their high scores out of ten when asked to rate this component of the program, and positive feedback provided by 63% of children. A child in ART Legends liked “that we got to talk together” while a KONTAKT™ participant felt they “got to know how everybody was feeling so if a person was a bit stressed, you could go easy on them.” Some appeared not to remember the specific opening and closing round activities as they reported enjoying the closing round because they “got to go home”, rather than reflecting on the actual closing ritual. Parents in both groups reported positively on the opening round:

I thought it was good. The introduction, I was there for the introduction. Excellent. They explained what the sessions were to be about to the kids, they sat them down calmly, they had it all up on the board. (Parent-ART Legends)

I think it was good because it focused on each child. And they all spoke. So that was something that they also have to get used to - they’re not used to having the attention on themselves, and I think that the staff really got to know them individually. They knew them inside out by the end of it. (Parent-KONTAKT^TM^)

Some children (24%) in the ART Legends group felt that the opening round went on “too long” and it was “a bit boring” whereas the negative opinions expressed by children in KONTAKT™ related more to the nature of the activity:

Well, yes, I did say my name. But still, I kind of didn’t want to talk about my feelings with other people, because I’m not kind of interested. Well, I guess it’s because I just get a little bit nervous when I start something new. And then it can take a while to get used to it. (Child-KONTAKT^TM^)

##### Activities

The activities in KONTAKT™ were generally regarded positively by the children, as reported by both parents and children. Children frequently reported charades as their favourite activity, saying “it was fun, because I like acting”.

I think he liked the activities. He liked the fact that it was a game and it’s like, you know, like a lot of the therapy services, when they’re put into games, they don’t realise that they’re learning the skills that they need, but in a fun, positive way. (Parent-KONTAKT^TM^)

Some children in KONTAKT™ (28%) reported the games were “boring” and that they didn’t enjoy the ones that were more difficult (e.g., “I really didn’t like the treasure hunt, like the treasure was pretty hard to find”). Children in the KONTAKT™ group suggested the addition of more games, “more variety”, and a “class pet” such as a dog or lizard would have improved their experience in the program.

Despite there being a range of activities offered in the art program, children reported doing “whatever I wanted to do” and building “whatever we like”. Parents of children in the ART Legends group reported that facilitators “adjusted the program”, ensuring children had opportunities to engage in activities aligned with their interests, which was generally perceived positively. For example, one child did “drawing every week” and another “did paper planes just about every session.” However, some parents were “disappointed” their child “didn’t try a few more things”. One child suggested “more computers so I could animate” would have improved their experience of the ART Legends program.

##### Discussion round

Themed discussions were unique to the KONTAKT™ group, with children more often reporting positively (61%) than negatively (44%) on this component of the program. Positive aspects centred around learning, with children reporting being able to “learn a little bit more”, “learn other people’s perspectives” and “learning things about socialising and other stuff.” Some children reported finding the discussions “boring” or “too basic” and sometimes “people interrupting” was an issue. Parents had limited insight into this component of the program, but commented that they thought the themes were “very good topics, things that would definitely help them” and mostly “appropriate for their age”. One parent commented that it was difficult for their child to participate in the themed discussion on social media as “some of the things he has never gone through…the social media, I mean, we haven’t exposed him to those things.” There were mixed opinions of the discussion on autism with some parents reporting the children “didn’t like it” and one parent reporting their child “sat under the table hiding, like she was covering her ears.” However, another parent perceived this topic positively, reporting:

One of the weeks was talking about my autism. It was one of the topics. I thought that’s good for him to have more – to talk about that a bit more of like, come up with ideas of how I can talk about my autism. (Parent-KONTAKT^TM^)

##### Show and tell

Show and tell was a component of the ART Legends program, providing children with the opportunity to share their artwork with the group. More than half of the children in the ART Legends group (59%) provided positive feedback about this component of the program. Children enjoyed telling “each other about what we made” because “people could ask questions and see what you’ve made”. Similarly, half of parents (50%) expressed positive opinions of this component of the program.

It’s good social skills building, because a lot of the time they’re too shy to actually talk. Well, stand up in front of a crowd of people and talk because that’s overwhelming because everyone’s looking at you. Yeah, so good experience, and it helps at school as well. (Parent-ART Legends).

Negative comments about the show and tell component mostly related to time constraints, with one child stating: “the vast majority of the time we never got to do it because we never had time.” Similarly, a parent expressed “they never had time to do it. Most people weren’t finished by the end of the session”.

##### Homework

Feedback about the homework component of KONTAKT™ was more negative than positive for both parents and children. More than half of the children (56%) expressed negative opinions, including “it felt like a waste of time” and “I didn’t really want to do them”. Similarly, some parents expressed the homework was difficult for their child to do.

Yeah, so writing, writing and reading are both quite challenging for him. And his executive functioning is quite low. So, to actually, you know, think through a mission, and then to work through all the steps in that, I think it was just a bit too much for him. (Parent-KONTAKT^TM^).

Some children did, however, express positive thoughts relating to the homework, saying it was “building me higher and higher in social skills” and “I could learn from my mistakes, and I know what I’m doing, and achieve goals”.

##### Snack time

Children generally enjoyed the snack break for “the food and you got to talk to people”, although they had different preferences for snacks, with some enjoying the popcorn offered and others forgoing the snack as they “didn’t like the flavour” available. Although some parents acknowledged the snack time as an opportunity to socialise “naturally” and “play games and almost put into a practical way of using the things that they learn during the meetings”, others viewed it as just an opportunity for their child to eat something between finishing school and going home for dinner.

##### Excursion

The excursion was an element of KONAKT™ only. Many parents (65%) of children in KONTAKT™ perceived the excursion positively, with more than half of children (56%) also reporting positive experiences. The excursion enabled children to practice skills such as deciding what they wanted to eat, ordering their own food, making requests (e.g., for napkins or sauce), and handling money. One child described the experience of ordering their food:

Well, one, we were expected to go up and order by ourselves, which is a big move for me. If I can avoid doing it, I will…I had to. Most of it, I had [sibling] with me, but then they said, ‘Oh, we don’t have this.’ I ordered vanilla slice at the time, but they didn’t have any. So, I had to go, ‘Okay, that’s okay. We can just make it caramel.’ (Child-KONTAKT^TM^)

Parents appreciated the opportunity for their child to practice skills such as ordering their own food “without me there whatsoever, how it actually happens in real life”. One parent described the excursion as “a huge success”.

##### Cleaning up

Putting away the art and craft supplies was specific to the ART Legends program. Parents generally perceived this as “important” as it was “good that they had to take responsibility for their own things and for helping each other.” However, some children were less enthusiastic about this component as they “normally haven’t finished what I was doing” or “thought it was boring”.

##### Moderating a session

Children in the KONTAKT™ group were given the opportunity to moderate a session themselves, choosing the activity and topic of discussion. More than half of children (56%) expressed positive experiences of this, saying “I got a feeling of what it’s like to lead the group” and “I got to choose what happened”. One child said the worst part about this component was that they “only got to do it once”.

### Person-related factors

Person-related factors captured any changes observed by parents and reported by children following participation in the SSGPs. These were categorised under the subthemes of relationships, communication, self-confidence, factors of emotionality, and awareness and knowledge.

#### Relationships

Conflict management skills included compromising and managing or resolving conflicts with others. Overall, the percentage of participants who reported improvement in these skills was similar between groups (K: 28%; A: 24%). KONTAKT™ participants more frequently reported improvement in conflict resolution skills (K:28%; A: 18%). For example, one parent commented: “He was getting really angry with his siblings. And now, even if the other two siblings are fighting, he’ll try and help them resolve it.”

Cooperation skills encompassed following rules and offering or accepting help. Only one child in the KONTAKT™ group reported being better at following rules. For offering and accepting help, participants in ART Legends (41%) reported improvements in offering or accepting help more often that those in KONTAKT™ (17%).

He actually got an award at school for helping out one of the year six kids who had a broken a bone in one of his foot and he had to wear this cast. You know, one of those moon-boot things, for like six weeks. And C took it upon himself to help this kid up the stairs, get down the stairs and move around the playground and stuff like that. So he actually he got an award for it. (Parent-ART Legends)

Initiating social behaviour included meeting new people, making new friends, and arranging to catch up with other people. Children in both groups reportedly improved in making new friends, although those in KONTAKT™ slightly more (56%) than those in ART Legends (41%). One child reported that “before [KONTAKT™] I struggled to try and make friends and after, so when I finished it, I made friends a lot more easily” while another in KONTAKT™ reported “learning to get my friendships to last, because people kind of trusted me a bit better.” Overall, improvement in initiating social behaviour was similar for both groups (K: 72%; A: 71%).

He became really good mates with one of the little kids. Like, we’re going to try and, we’ve swapped phone numbers, and we’re going to try and catch up again. (Parent-ART Legends).

Interpersonal interactions included attending social events, hanging out with friends, informal relationships and joining a group. The most frequently reported improvement in this area was joining a group, with participants in both groups reporting similar improvement (K: 44%; A: 41%). Overall, more children in KONTAKT™ (61%) reportedly improved in interpersonal interactions than those in ART Legends (53%).

#### Communication

Foundational skills included listening to others and taking turns. Children in both groups reportedly improved in this area, with a higher proportion of those in the ART Legends group. For listening to others, 41% of children in the ART Legends group were perceived to have improved, compared to 33% in KONTAKT™. Taking turns was also more frequently reported as an improvement for those in the art group (53%) than KONTAKT™ (39%).

Non-verbal communication included recognising emotions and expressing emotions. Half of all KONTAKT™ participants (50%) reported improvement in this area compared to 41% of ART Legends participants. For overall verbal communication, which included expressing emotions, starting and maintaining conversation, others understanding what they mean, and ordering something, for example, at a cafe, more children in KONTAKT™ (56%) reportedly improved than in ART Legends (35%). However, children in both groups improved similarly in starting conversations (K: 33%; A: 35%).

But he is getting better at it, like initiating a conversation. Like he had one of his mates come over for a sleepover, and listening to them talk in his bedroom was really quite comforting. (Parent-KONTAKT^TM^)

#### Self-confidence

Skills in self-confidence encompassed assertiveness, self-worth and self-confidence while socialising. More children in KONTAKT™ (44%) reportedly improved than those in ART Legends (35%).

I can just see that he, I mean, he’s always so motivated for Thursdays, when the classes were, so that he had someone, you know, some other people see and he he’d say, Oh, I want to meet new people. Because he’s, and these people were like him. I think that helped. I think that gave him confidence. Knowing that there are kids like him. (Parent-KONTAKT^TM^)

#### Factors of emotionality

Factors of emotionality included such skills as emotion regulation, managing emotions, loneliness, stress, and tolerating social situations. Only one participant in ART Legends reported improvement in this domain compared to 44% of KONTAKT™ participants. Managing emotions (39%) and loneliness (33%) were the most frequently reported areas of improvement for those participants.

#### Awareness and knowledge

Awareness and knowledge included social awareness such as understanding social rules and social situations, as well as understanding what others mean in interactions. Overall, more participants in the KONTAKT™ group (33%) reported improvement in awareness and knowledge than in ART Legends (24%). However, more children in ART Legends reportedly improved in understanding social rules, specifically, than those in KONTAKT™ (17%).

### Factors affecting participation

Factors affecting participation were classified as facilitators or barriers. However, this varied depending on the participant’s perception. While many parents denied the need to incentivise their child’s participation in either group, some parents (35%) provided special food (e.g., doughnut, milkshake, ice cream) before or after the session, with parents acknowledging this “could have been incentivising, yeah, but there was no other sort of encouragement.”

Some parents (27%) reported the timing and location of the sessions enabled an easy transition between school and the activity, while others (46%) reported these aspects as barriers due to travel distance and clashes with other activities. For participants in ART Legends, an interest in art and craft was considered a facilitator, with children being motivated to participate because they were “very interested in doing it because of the art aspect.” Support workers or family members such as grandparents, who assisted with transport to and from either group, also enabled participation for children whose parents were unable to take them or who had siblings enrolled in activities at the same time.

It was just a bit of bad luck that it fell on Thursday afternoons because his brother plays basketball, and I’m the coach. So, there was just no way that I couldn’t not do that, you know? (Parent-ART Legends)

Having other children to consider was identified as a barrier to participation by parents from both groups as often they had other activities or responsibilities to manage. Similarly, some parents had to “leave work early” or take their child out of school early, or make alternative arrangements, to ensure they arrived at the group on time. COVID- 19 was also identified as a barrier by 24% of parents, with some children being unable to attend their group due to infection of either themselves or another family member.

For children in the art group, one parent mentioned that the program “started in one building and then they had to transfer buildings and even when he first started in the original building, they moved rooms…it wasn’t the same place, the same room, the whole time”, which presented a barrier to their child’s participation. Parents suggested that children in the art group required more “scaffolding and support” to enable their participation in a range of activities. One parent reported that their child did not “identify” with autism and therefore attending a group specifically for children on the autism spectrum may have been a barrier to their participation. Children in the ART Legends group reported the “leaders kept changing” which both children and parents perceived as a barrier to their participation due to lack of communication about this change preventing adequate preparation.

### Research-related factors

Participants were asked about their experience participating in research, which included sharing their opinions of the measures used to evaluate the SSGPs. Some parents of children in the KONTAKT™ group (29%) reported that the goal setting process was easy to do while others (35%) reported that it was difficult for their child and could have been improved by providing “more visuals”, “more prompting” and “some more examples”. Some children (28%) also reported it was “hard” or “complex” for them to set the goals.

A measure, based on the ICF [39], was developed for use in this study. Many parents of children in the KONTAKT™ group reported this was difficult to understand and 22% of children in that group agreed it was not easy to do due to the large number of questions, “confusing” questions, and language used (e.g., weakness, strength). Similarly, some parents (41%) reported the online surveys were difficult to do due to the language used and they were repetitive and time consuming. Some parents found the weekly survey sent via text message was “annoying” and a “bit confusing” but “much easier than the other questionnaires”.

Currently intended only for children on the autism spectrum, parents whose children were in the KONTAKT™ group were asked to share their opinions of including neurotypical peers in the groups. Overall, 47% of parents were in favour of including neurotypical children while 29% would prefer the groups to remain specifically for children on the spectrum. Parents identified a range of potential positive and negative outcomes of including neurotypical children. Some parents suggested that neurotypical children could also benefit from improving their social skills and that including children on the spectrum in groups with same-aged neurotypical peers could facilitate sharing of knowledge and skills. Other parents suggested that neurotypical peers could be included as role models or “mentors” for children on the spectrum. It was also suggested that including neurotypical peers could improve “social awareness” and understanding of autism among neurotypical children. However, some parents were concerned that including neurotypical peers would reduce the effectiveness of the program and prevent children on the spectrum from “feeling free to really share openly”. One parent summarised this sentiment:

I think it would have been a negative. I think it was really important that it was just kids with autism, or, you know, autism and [co-occurring] conditions, or, or kids with ADHD. Whatever it is, that was really important, just because their issues are very different from kids who are neurotypical, they don’t experience the same concerns and, you know, stresses in their lives. (Parent of KONTAKT^TM^ participant)

## Discussion

This study set out to explore the lived experiences of participants involved in two different types of social groups for children (aged 8 to 12 years) on the autism spectrum: KONTAKT™, a more explicit social skills training program, and ART Legends, a less structured social art group fostering opportunities for more implicit social learning. The purpose of this study was to contrast participants’ experiences, identifying the factors affecting participation in social groups and determining which elements of the groups were perceived as useful and/or enjoyable for the children and families involved in them. Although previous studies have qualitatively explored parents’ and adolescents’ experiences of KONTAKT™ [18, 19], this study contributes additional and novel perspective of children and families involved in the intervention (KONTAKT™) group or the active control (ART Legends) group.

The findings of this study suggest that children in both groups demonstrated improvements in their social skills. This is not surprising, given the strong body of quantitative evidence supporting the efficacy of SSGPs in contributing to improved friendships, reduced loneliness, and greater social competence [13, 14]. The experiences of the younger children included in this study echo those of adolescents previously interviewed about their participation in KONTAKT™ [18, 19], suggesting that SSGPs are appropriate for both children and adolescents. Although children in the ART Legends group were not explicitly taught social skills, outcomes reported by parents and children were similar to those discussed by participants in the KONTAKT™ group. More informal social groups such as the purposely designed ART Legends group used in this study enable children to learn social skills by exposing them to different social experiences in a group context and can promote generalisability of skills [40]. However, SSGPs such as KONTAKT™, employing a blended approach of informal social learning and explicit social teaching, may have a greater influence on factors of emotionality, with the findings of this study suggesting children in the intervention group experienced improvements in managing their emotions and loneliness, whereas those in the control group did not report change in this area.

The results of the broader RCT evaluating the efficacy of KONTAKT™ in comparison to the active control group, ART Legends, found that children made progress towards their personally meaningful social goals, regardless of which group they participated in (Afsharnejad B, Lee EAL, Hayden-Evans M, Fridell A, Coco C, Johnson M, et al: Which should I attend: KONTAKT Social Skills Group Program or ART Legends Social Group? A double-blinded randomised controlled trial, in preparation). However, children who attended KONTAKT™ reported significantly improved quality of friendship at follow-up (3 months after the intervention) in comparison to those who participated in ART Legends (Afsharnejad B, Lee EAL, Hayden-Evans M, Fridell A, Coco C, Johnson M, et al: Which should I attend: KONTAKT Social Skills Group Program or ART Legends Social Group? A double-blinded randomised controlled trial, in preparation). The findings of this qualitative study suggest that children in both groups made new friends, suggesting that SSGPs with varying approaches can promote the initiation of friendships, but explicit social groups may have greater long-term benefits for children on the spectrum as they specifically teach children the skills required to foster ongoing friendships in addition to providing initial opportunities to meet new people.

Overall, factors affecting participation in either of the SSGPs were similar. The unique interaction between factors largely determined if the influence on participation was positive or negative. Logistical factors such as the timing and location of the groups were frequently cited as factors affecting participation, suggesting that these need to be carefully considered when designing SSGPs for children on the spectrum. Given the barriers presented by travel time, distance, and schedule clashes, future research should focus on evaluating the feasibility of SSGPs embedded in the school curriculum [15], minimising the need for children to attend external sites. There is evidence to suggest that both a version of KONTAKT™ (SKOLKONTAKT™) and other manualised programs have been successfully implemented in school contexts, resulting in positive outcomes for both students and teachers [15, 41]. The school environment presents an opportunity for children to learn and practice new skills in real-word situations, promoting generalisability of these skills beyond the clinical context [42]. In addition, parents appreciated the extended length of the intervention period in the current study, reporting that the more frequent sessions during school terms were more beneficial for their child than one-off programs conducted during the school holidays. This aligns with the findings of a Swedish RCT comparing different variants of KONTAKT™ that concluded the extended version of the program (24-weeks) had greater long-term benefits than the shorter variant (12-weeks; Jonsson et al., 2019).

Many of the children in the ART Legends group were motivated to participate due to their existing interest in art. For children on the spectrum with specific interests, incorporating these in interventions can have a positive influence on attention, motivation and performance [43]. In addition, interest-based SSGPs may facilitate social interactions by bringing together groups of children with similar interests and abilities [44]. Among the suggestions for improvement of the SSGPs was the inclusion of a group pet. There is a growing body of evidence supporting the influence of animal-assisted interventions on social interaction, language and communication, emotions, and behaviour of autistic individuals [45]. While the inclusion of an animal such as a therapy dog may not be suitable for all children, there are some studies supporting the use of dogs in social skills training for children on the spectrum, with those children who interacted therapeutically with a dog demonstrating a greater reduction in autistic traits than those who participated in traditional social skills training [46].

Aligning with the findings of the Australian qualitative study exploring adolescents’ experiences of KONTAKT™ [18], parents in the current study had mixed opinions regarding the integration of neurotypical peers in SSGPs for children on the spectrum. While some expressed it could present opportunities for neurotypical children to improve their awareness, understanding and acceptance of autism, others suggested that it was important for children on the spectrum to have a safe space to be themselves and connect with other children who can relate to their situation. For autistic adults, connecting with other autistic individuals can contribute to building a sense of belonging, increasing confidence, and providing companionship, ultimately leading to a sense of autistic community [47]. Autism-specific groups for adolescents provide similar benefits, boosting self-esteem, improving health and wellbeing, and contributing to improved social relationships and interactions [48]. While there is limited research exploring the benefits of autism-specific groups for younger children, the findings of this study indicate that parents appreciated the opportunity for their child to participate in a group where their autism was celebrated and understood by the other group members, suggesting connection with autistic community may be as important for children as it is for adolescents and adults.

There is currently a shift occurring in autism research, away from a problem-focused perspective and towards neurodiversity and active participation of the autistic community [49]. The concept of ‘nothing about us without us’ remains a strong theme among research exploring the perspectives of the autistic community on research priorities [49, 50], highlighting the critical need to incorporate the lived experiences of autistic individuals and their families in the research that ultimately impacts them. This qualitative study was designed to capture the perspectives of children on the spectrum and their parents, enabling them to share their experiences of the different types of SSGPs, and enhance the quantitative findings of the broader RCT (Afsharnejad B, Lee EAL, Hayden-Evans M, Fridell A, Coco C, Johnson M, et al: Which should I attend: KONTAKT Social Skills Group Program or ART Legends Social Group? A double-blinded randomised controlled trial, in preparation) through their narratives. Key considerations for designing and implementing SSGPs for children on the spectrum, from the perspective of children and their caregivers, are summarised in Table 4 below.

**Table 4:** Key considerations for designing and implementing social skills group programs for children on the autism spectrum

Key considerations	Example/s
Time of group sessions	Minimise disruption to school and work schedules
Location of group sessions	Proximity to school/home; consistency of location
Snack preferences	Offer variety; alternative flavours/options
Age range	Reducing the age range within groups
Group size	Ensuring sufficient children to make a ‘group’ in the event of illness etc
Activities aligning with interests	Incorporate special interests/strengths; enhance learning through games or enjoyable activities
Therapy animal	Incorporate therapy dog/animal in some sessions
Weekly text/email to parents	Summarise content covered each week
Autism-specific	Promote sense of belonging and community
Routine and structure	Repetitive agenda; familiar activities
Responsive to participants’ needs	Allow flexibility to support individual needs and interests of group members

### Limitations

While this study provides valuable insight into the experiences of children and families who participated in either of the two different types of social groups for children on the spectrum, there are limitations that must be addressed. Less than half of eligible participants (45%) accepted the invitation to be interviewed. It is unclear whether the experiences of the children and families who declined to participate would have differed from those reported, or what their reasons were for not participating in the interview. Although parents and children reported improvements in the child’s social skills following the intervention period, it is impossible to conclude whether these gains were purely the result of participating in either KONTAKT™ or ART Legends, or if these could also have been influenced by other internal (e.g., developmental progression) or external influences (e.g., school, therapy). In this study, both children and their caregivers were interviewed, however, the depth of data provided by the children was limited in comparison to the caregivers. While researchers implemented strategies to facilitate communication with the children (e.g., visual aids), difficulties communicating one’s own thoughts and feelings are characteristic of autism, especially at a young age, and may have limited the true expression of the children’s experiences. In addition, the study occurred during the COVID- 19 pandemic, which presented both a barrier to participation in the intervention and data collection processes. Interviews were conducted online to prevent the spread of infection, which may also have contributed to challenges in communication between the researchers and participants.

## Conclusions

This study contributes to the growing evidence base supporting the use of SSGPs to improve social communication and interaction skills among children on the autism spectrum. Using a qualitative approach to explore the perspectives of children and caregivers involved in two different types of social groups, this study suggests that both more structured, explicit and more implicit approaches can have positive outcomes for children on the spectrum, as perceived by their parents and the children themselves. However, there are both barriers and facilitators to participation that should be taken into consideration when designing SSGPs for children on the spectrum. Although participants’ overall experiences of either group were predominantly positive, feelings towards individual aspects of the groups were highly subjective and dependent on a range of personal factors.

## Supplementary Information

Supplementary Material 1

## Data Availability

The data supporting the findings of this study are not publicly available due to privacy and ethical restrictions.
