# Supplementary material for: “I made friends a lot more easily”: children and families’ experiences of social group programs for children on the autism spectrum
Source: BMC Pediatr. 2025 May 3;25:353. doi: 10.1186/s12887-025-05686-6 (PMC12048955; doi:10.1186/s12887-025-05686-6)
Supplement: Supplementary file 1 — Supplementary Material 1 [file 12887_2025_5686_MOESM1_ESM.pdf]

Post KONTAKT® Interview

Parents

Hi, I hope you and your child have enjoyed attending the KONTAKT® social skills program.

We know each person's experience with this program is different. Therefore, we would like to ask you a series of questions to understand how the program went for you and your child and how you both felt about it. These questions will cover the structure and the content of program and your perceptions of it. Please answer each question to the best of your ability.

STRUCTURE

Here we ask you a few questions about the structure of the KONTAKT® program.

1. What do you think about the number of sessions? Was there enough? Was there too many?
2. Do you think you received enough information about the program prior to it starting?
3. Do you think you received enough information about your child's progress throughout the course of the program?
4. What do you think about the number of *parent sessions* in KONTAKT®? Was there too many, too few or just enough?
5. What do you think about the number children in your child's group? Was there too many, too few or just enough?
6. What do you think about the age range of the children in the group? How did your child feel about it?
7. Did the weekday and the time of the sessions work for you?
8. How did your child travel to the groups (e.g. by bus, I drove my child, walked, …)?
9. Did your child come to groups straight from school?
10. Did you/ your partner stay at the Autism Association centre while your child was at the group? If not, generally what did you do during the session times?
11. Who picked your child from the centre?
12. Did you/your partner have to give up work or other responsibilities to pick up your child from the KONTAKT® sessions?
13. Did you buy gifts for/pay your child to participate in the KONTAKT® session or to do the missions? Did you use other incentives to encourage them to participate in KONTAKT® or complete their missions?
14. How much time did you spend working with your child on the KONTAKT® missions? Was it hard for your child to complete the missions? If so, why?

CONTENT

As you have seen in your activity book, the KONTAKT® group follows the same weekly agenda. I would like to ask for your feedback on each of the aspects of this agenda, as far as you feel able to comment.

1. What do you think about the introduction and closing rounds on the KONTAKT® agenda? How do think your child felt about them?
2. What do you think about the missions/homework assignments included in KONTAKT®?
3. How do think your child felt about doing these?
4. Did you help your child with the missions? Why/why not?
5. Did you child have any issues completing the missions/homework assignments?
6. What do you think about snack time in the KONTAKT® agenda? How did your child experience this?
7. What do you think about the games and activities that were covered in the KONTAKT® sessions? How do think your child felt about them?
8. What do you think about the Discussion round in the KONTAKT® agenda? How do think your child experienced this?
9. What do you think about the Excursion? How do think your child experienced this?

EXPERIENCES

During KONTAKT® your child worked on different topics. These are listed below if you do not remember.

1. Do you think your child has now become better at some of these skills?
2. Do you think that after participating in KONTAKT® there are somethings that have become more difficult for your child?

- Recognising emotions in face
- Recognising emotions in body
- Initiating a conversation
- Managing conflicts
- Managing stress
- Joining a group
- Understanding Social rules
- Compromising
- Saying No
- Resolving conflicts
- Going out to a café
- Find new friends
- Expressing emotions in your face
- Expressing emotions in your body
- Meeting new people
- Social situations
- Self confidence
- Attending social event
- Calling someone on the telephone
- Taking turns
- Listen to others
- Offering or accepting help
- Setting a meeting or date
- feeling lonely

- Understanding what others mean by what they are saying
- How to get others to understand what you are saying

3. Which part of the program do you think has been most important for your child's social development?
4. In addition to the improvements in social skills, did participating in the KONTAKT® program influenced your child in any other way?
5. Has participating in the KONTAKT® program had any negative effects on your child? Has anything gotten worse as a result of their participation in the KONTAKT® program?
6. During the time your child was participating in KONTAKT®, how has your life been? Has there been any events that might have affected their behaviour during their participation in this program?
7. When you reflect on your child's participation in the KONTAKT® program, what do your child's experience has been? Overall was participating in KONTAKT® a positive or negative experience?
8. Is there anything you think the KONTAKT® trainers should have done more of?
9. Is there anything you think the KONTAKT® trainers should have done less of?
10. That is all on the questions, but please feel to share any information you think relevant concerning your child's participation in the KONTAKT® program.
11. How do you feel about having someone who is <u>not on the autism spectrum</u> in the group?
12. If your answer to the previous question is positive, which group age do you prefer, **primary school** OR High school?

EXPERIENCES - MEASURES

You and your child also completed some questionnaires online and attended some online or face to face assessment sessions (online or face to face) where your child participated in some activities with one of the researchers.

1. Everyday activities:

Child version

Watching
(a)

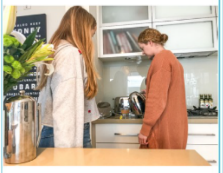

d110

How much difficulty do you have **watching people**

d110

Do not understand		Weakness					Average			Strength	
	0	1	2	3	4	5	6	7	8	9	10
	Can't do	Very difficult		Difficult		Sometimes difficult		Easy		Very easy	

Parent version

International Classification of Functioning for Autism

The following questionnaire asks you to rate your child's ability to do a range of everyday activities.

Each item is scored according to how well you think they CAN do an activity (given necessary support and encouragement) and how well they usually DO the activity.

Note: If your child is unable to do an activity, please score that item "0".

Note: If an item is not relevant or age-appropriate, you may score it as "Not Applicable".

Note: If you are unsure, you may score it as "Not Known".

1. LEARNING AND APPLYING KNOWLEDGE

1-1. How much difficulty does your child have with using the senses to gather information:

Watching - persons

	Weakness						Average			Strength	
	0	1	2	3	4	5	6	7	8	9	10
CAN	○	○	○	○	○	○	○	○	○	○	○
DOES	○	○	○	○	○	○	○	○	○	○	○

a. How easy was this activity for your child?
b. Do you feel your child understood the questions?
c. How can we improve this survey?

2. Weekly texts:

a. What was your/your child's experience with the weekly text?
b. How easy was this activity for you/your child?
c. How can we improve this survey?

3. Goal setting:

a. What was your child's experience with the goal setting?

b. How easy was this activity for your child?
c. How can we improve this goal setting process?

That is all of the questions I wanted to ask. Is there anything else you would like to share about your experience of attending the KONTAKT® program?

Thank you for being involved in our study!

Post KONTAKT® Interview

Children

Hi, I hope you have enjoyed attending the KONTAKT® social skills program

I have not attended KONTAKT® and I have no information about what happened in your group or what you, your group mates or trainers did during the KONTAKT® sessions. That is why we are going through this interview so that you can let me know all about it.

STRUCTURE AND CONTENT

We are going to talk about some aspects of KONTAKT®. This includes four questions. I am going to ask you to:

1. Tell me what happened.
2. Say how you felt about them/experienced them.
3. Say what was good about them.
4. Say what was less good about them.

1. **Opening Round:** At the beginning of each of the KONTAKT® sessions you said your name and choose an emotion from a list and shared with the group.

   1. Can you tell me what happened (describe it)? (provide some examples)
   2. On a scale of 1 to 10 how did you feel about it?

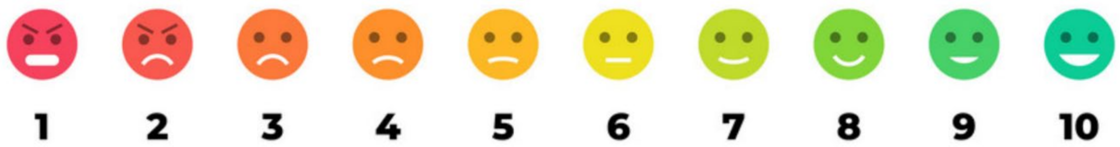

   3. Can you tell me what was good about it?
   4. Can you tell me what was less good about it?

How do you you feel?

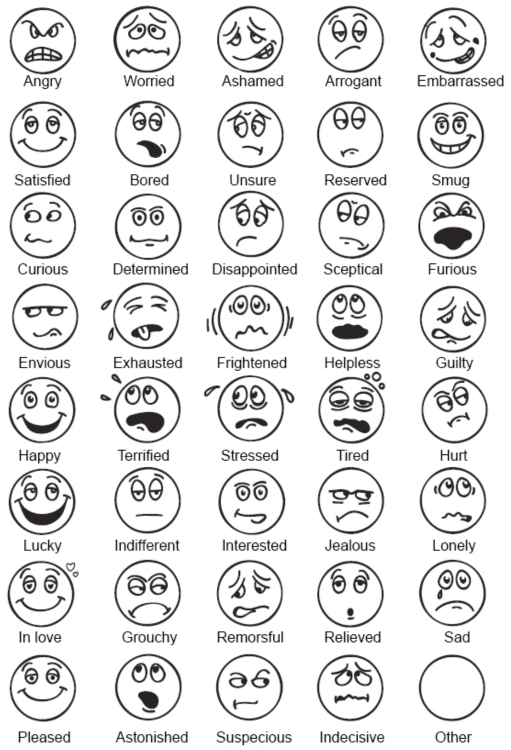

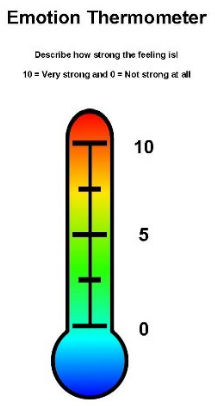

2. **Group Activities and Games:** Each session had some group exercises or games.

5. Can you tell me what happened (describe it)? (provide some examples)
6. On a scale of 1 to 10 how did you feel about it?

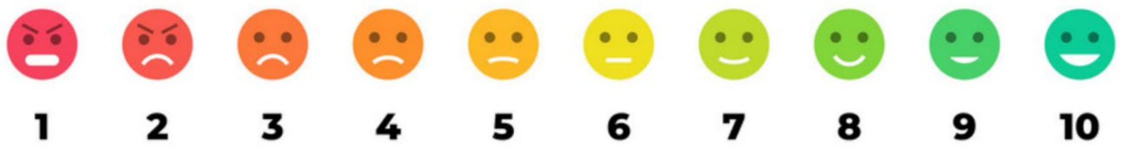

7. Can you tell me what was good about them?
8. Can you tell me what was less good about them?

Here is the list of games and activities covered in KONTAKT®. <u>You might have only done some of them</u>.

- The blinking game
- Treasure hunt
- Spin the bottle
- Emotions game
- Charades
- Role play
- Fruit basket
- Emotion videos
- Alphabet Sentences
- Heads up!
- 20 Questions
- Advanced treasure hunt
- What has changed
- Square breathing
- Whispering game
- Head band
- Question ball

9. Which ones did you like? Why?
10. Which ones didn't you like? Why?

3. **Snack Break:** During these sessions there was a time when you could have a rest and eat something, right?

11. Can you tell me what happened (describe it)? (provide some examples)
12. On a scale of 1 to 10 how did you feel about it?

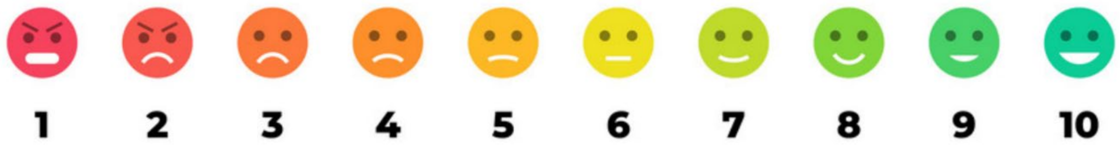

13. Can you tell me what was good about them?
14. Can you tell me what was less good about them?

4. **Group Discussions:** Each session involved group discussions. Here is the list of some of the topics you discussed during the KONTAKT® sessions. <u>You might have only covered some of them in your KONTAKT® sessions</u>.

- Friendship
- Social rules
- Compromising
- Saying No
- Resolving conflicts
- Going out to a café
- Moderating a session
- What is ASD
- Expressing criticism
- Calling someone
- Taking turns
- Stress
- Offering or accepting help
- Setting a meeting or date
- Handling challenging situations on social media
- Connecting through social media
- feeling alone and being teased and/or bullied
- Taking social initiatives (e.g. contacting a person you don't know)

15. Can you tell me what happened (describe it)? (provide some examples)
16. On a scale of 1 to 10 how did you feel about it?

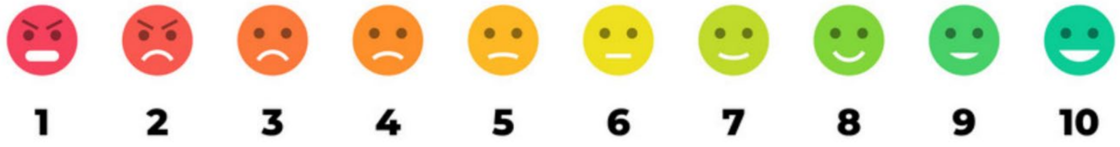

17. Can you tell me what was good about them?
18. Can you tell me what was less good about them?

5. **Closing Round:** At the end of each session there was a routine for ending or closing the session, do you remember that?

19. Can you tell me what happened (describe it)? (provide some examples)
20. On a scale of 1 to 10 how did you feel about it?

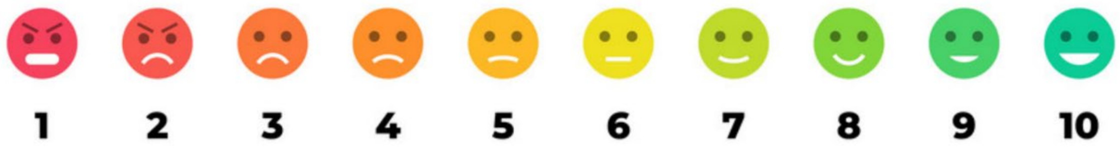

21. Can you tell me what was good about them?
22. Can you tell me what was less good about them?

6. **Missions:** Every session you had to complete a mission, right? Below is an example.

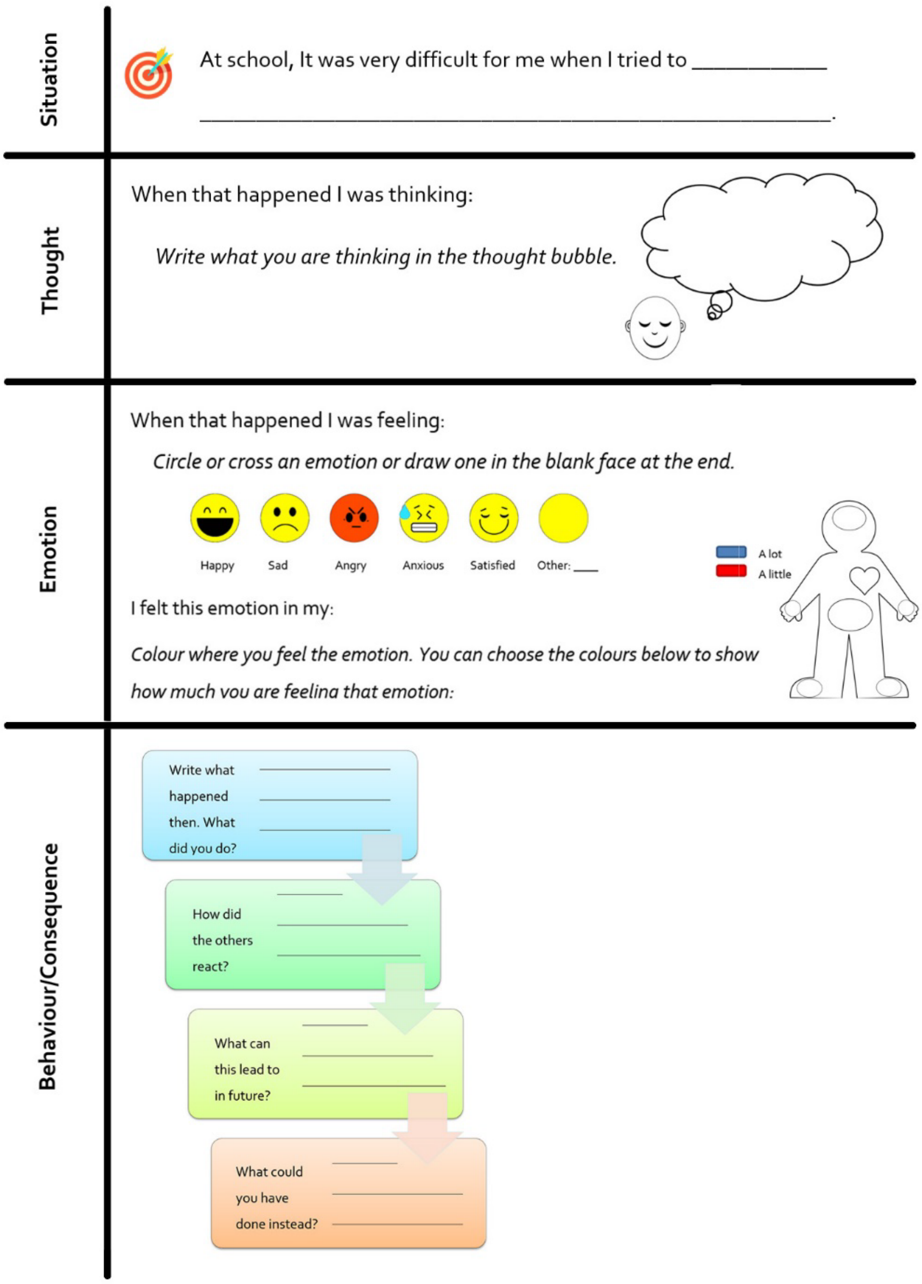

23. Can you tell me what happened (describe it)? (provide some examples)
24. On a scale of 1 to 10 how did you feel about it?

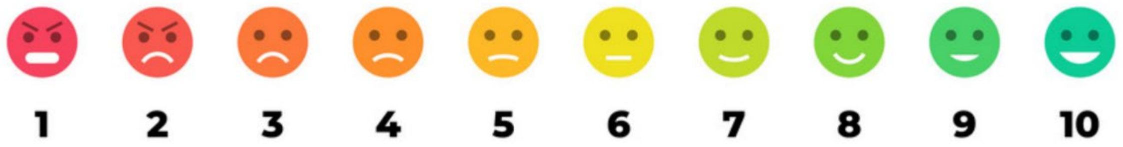

25. Can you tell me what was good about them?
26. Can you tell me what was less good about them?

7. **Moderating a discussion or activity:** During a session you and another group member had the opportunity to moderate some parts of a session.

27. Can you tell me what happened (describe it)? What did you do? (provide some examples)
28. On a scale of 1 to 10 how did you feel about it?

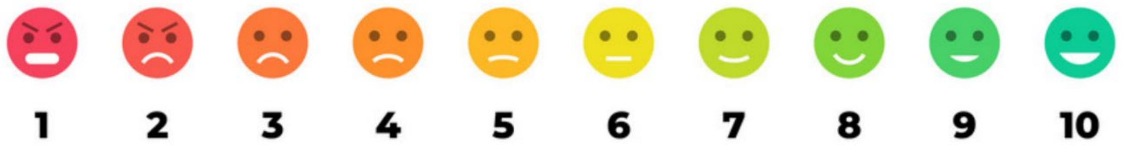

29. Can you tell me what was good about them?
30. Can you tell me what was less good about them?

8. **Excursion:** During a session you went to a café.

31. Can you tell me what happened (describe it)? What did you do? (provide some examples)
32. On a scale of 1 to 10 how did you feel about it?

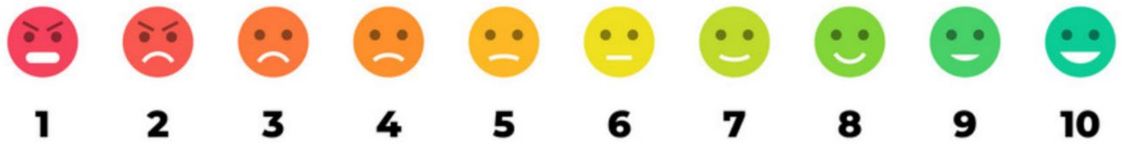

33. Can you tell me what was good about them?
34. Can you tell me what was less good about them?

9. **Number of people in the group:** What do you think about the number of people in your group? Was is there too many, or not enough?

35. Can you tell me about it (describe it)? (provide some examples)
36. On a scale of 1 to 10 how did you feel about it?

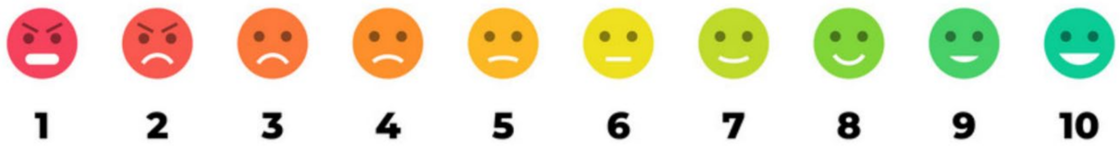

37. Can you tell me what was good about it?
38. Can you tell me what was less good about it?

10. **Group Members:** You had a few other people in the group. Did you like it? Did you make friends? (provide some examples)

39. Did you make friends? Can you tell me about it (describe it)? (provide some examples)

11. On a scale of 1 to 10 how did you feel about it?

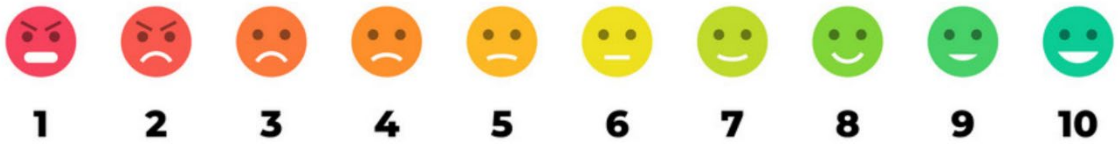

EXPERIENCES - KONTAKT®

The KONTAKT® program covers various social skills topics. There is a list below if you do not remember them.

A. Do you think by attending KONTAKT® you have become better at some of these skills? **Prompts**: If so, which ones? Provide some examples.

B. Do you think by attending KONTAKT® doing some of these skills have become more difficult for you? **Prompts**: If so, which ones? why do you think this is? Provide some examples.

- Recognising emotions in face
- Recognising emotions in body
- Initiating a conversation
- Managing conflicts
- Expressing emotions on your face
- Expressing emotions with your body
- Meeting new people
- Social situations

- Managing stress
- Joining a group
- Understanding Social rules
- Compromising
- Saying No
- Resolving conflicts
- Going out to a café
- Making new friends
- Self confidence
- Attending a social event
- Calling someone on the telephone
- Taking turns
- Listening to others
- Offering or accepting help
- Setting a meeting or date
- Feeling lonely
- Understanding what others mean by what they are saying
- How to get others to understand what you are saying

C. Did attending the KONTAKT® program improve your social skills? **Prompts:** Can you tell me more about it? Why do you think this is?

D. Did attending the KONTAKT® program impact your life in any other way? (For example, did you stop/start doing something else to attend the KONTAKT® program?)

E. When you think back over the 16 KONTAKT® sessions, what do you think was your overall experience?

F. Was attending KONTAKT® overall a positive or negative experience?

G. Would you recommend KONTAKT® to other autistic kids?

H. In the KONTAKT® program was there anything you would have liked to have done more of?

I. In the KONTAKT® program was there anything you would have liked to have done less of?

EXPERIENCES - MEASURES

You also completed some questionnaires online and attended some online or face to face assessment sessions (online or face to face) where you participated in some activities with Bahareh (you might know her as “B”) or Maya. This includes four questions. I am going to ask you to:

a. Say how easy it was to do them.
b. Say what was easy/good about them
c. Say what was less easy/good about them.
d. Say how we can we make it better.

1. Everyday activities:

Watching
(a)

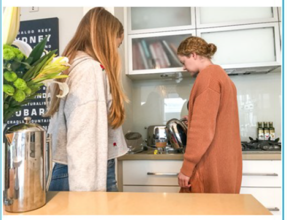

d110

How much difficulty do you have **watching people**

d110

Do not understand											
		Weakness					Average			Strength	
	0	1	2	3	4	5	6	7	8	9	10
	Can’t do	Very difficult		Difficult		Sometimes difficult		Easy		Very easy	

a. On a scale of 1 to 10 how easy was it for you to answer the questions?

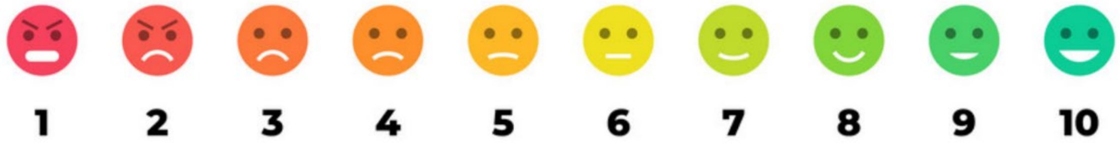

2. Can you tell me what was easy/good about answering this survey?

3. Can you tell me what was less easy/good about answering this survey?

4. Can you tell me how we can make this survey better?

5. Weekly texts:

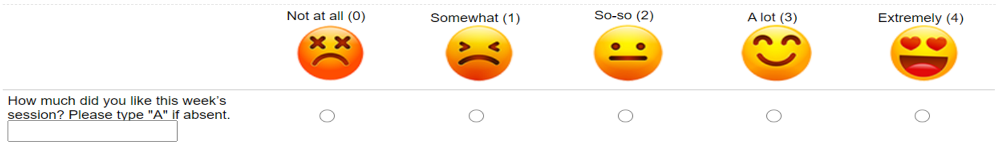

a. On a scale of 1 to 10 how easy was it for you to answer the questions?

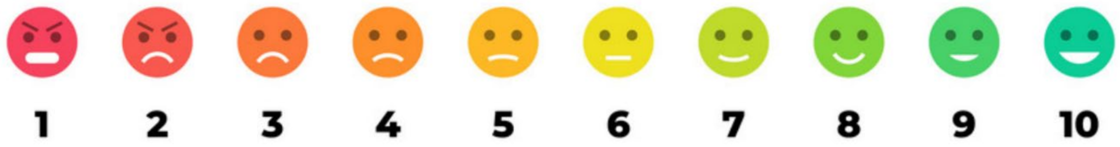

6. Can you tell me what was easy/good about answering this survey?

7. Can you tell me what was less easy/good about answering this survey?

8. Can you tell me how we can make the online survey better?

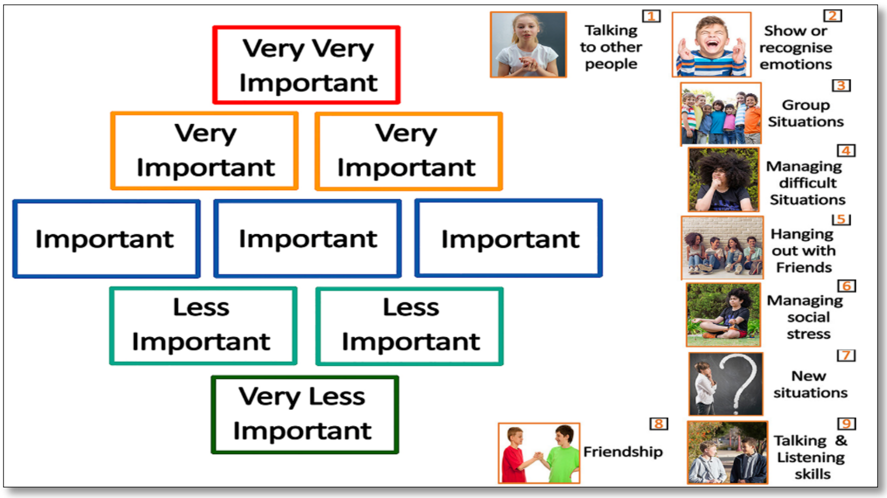

9. On a scale of 1 to 10 how easy was it for you to set your goals?

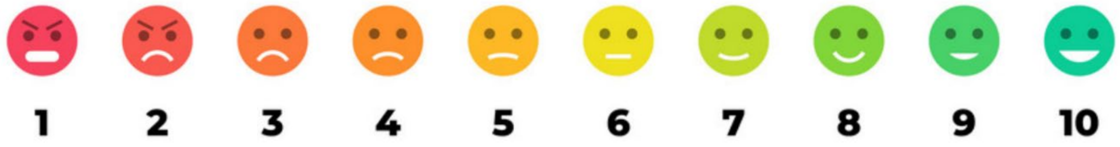

10. Can you tell me what was easy/good about setting your goal?

11. Can you tell me what was less easy/good about setting your goal?

12. Can you tell me how we can make the goal setting process better?

13. Online surveys:

Please write the name of your **best friend** in the box:

We want to know how you get along with your best friend.

We have some sentences that we would like you to read. Tell us how true the sentence is for your friendship.

After each sentence, there is a slide bar that goes from 1 to 5.

"1" means the sentence is probably "not true" for your friendship
"2" means that it *might be true*
"3" means that it is usually true,
"4" means that it is very true
"5" means that it is really true for your friendship.

Example
My friend and I play games and other activities with each other.

Not true Really true
1------2------3------4------5

Remember, there are no right or wrong ways to answer these questions, and you can slide the bar to any number you think is right. *Be sure to read carefully and answer as honestly as possible.*

Not true	Might be true	usually true	Very true	Really true
1	2	3	4	5

1. I do what I can to make sure my friendships will last for a very long time

These questions ask about how you are today. For each question, read all the choices and decide which one is most like you today. Only choose one answer for each question.

Example
Today I feel quite upset so I will tick this box.

Upset
o I don't feel upset today
o I feel a little bit upset today
o I feel a bit upset today
þ I feel quite upset today
o I feel very upset today

Now think about and answer the rest of the questions below

1. Worried
- I don't feel worried today
- I feel a little bit worried today
- I feel a bit worried today
- I feel quite worried today
- I feel very worried today

You may also have completed this survey.

In this survey, you will find statements people might use to describe their attitudes, opinions, interests, and other personal feelings.

Each statement can be answered TRUE or FALSE. Read the statement and decide which choice best describes you. Please answer every statement, even if you are not completely sure of the answer.

Read each statement carefully, but don't spend too much time deciding on the answer. Try to describe the way you usually or generally act and feel, not just how you are feeling right now.

Remember there are no right or wrong answers - just describe your own personal opinions and feelings.

	True	False
1. I get tired and need to rest more than other kids my age.	○	○
2. I usually like other kids even when they are very different from me.	○	○

a. On a scale of 1 to 10 how easy was it for you to answer the questions?

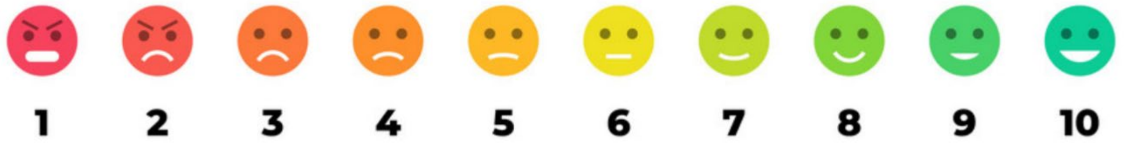

14. Can you tell me what was easy/good about answering this survey?

15. Can you tell me what was less easy/good about answering this survey?

16. Can you tell me how we can make the online survey better?

That is all of the questions I wanted to ask. Is there anything else you would like to share about your experience of attending the KONTAKT® program?

Thank you for being involved in our study!

Post ART Interview

Parents

Hi, I Hope you and your child have enjoyed attending the ART social program.

We know each person's experience with this program is different. Therefore, we would like to ask you a series of questions to understand how the program went for you and your child and how you both felt about it. These questions will cover the structure and the content of program and your perceptions of it. Please answer each question to the best of your ability.

STRUCTURE

Here we ask you a few questions about the structure of the ART program.

- What do you think about the number of sessions? Was there enough? Was there too many?
- Do you think you received enough information about the program prior to it starting?
- Do you think you received enough information about your child's progress throughout the course of the program?
- What do you think about the number children in your child's group? Was there too many, too few or just enough?
- What do you think about the age range of the children in the group? How did your child feel about it?
- Did the week day and the time of the sessions work for you?
- How did your child travel to the groups (e.g. by bus, I drove my child, walked, …)?
- Did your child come to groups straight from school?
- Did you/ your partner stay at the Autism Association centre while your child was at the group? If not, generally what did you do during the session times?
- Who picked your child from the centre?
- Did you/your partner have to give up work or other responsibilities to pick up your child from the ART sessions?
- Did you buy gifts for/pay your child to participate in the ART sessions or to do the missions? Did you use other incentives to encourage them to participate in the ART program or complete their missions?

CONTENT

As you have seen in your activity book, the ART program follows the same weekly agenda. I would like to ask for your feedback on each of the aspects of this agenda, as far as you feel able to comment.

1. What do you think about the introduction and Closing rounds on the ART agenda? How do think your child felt about them?
2. What do you think about snack time in the ART agenda? How did your child experience this?
3. What do you think about the Arts and crafts that were covered in the ART sessions? How do think your child felt about them?
4. What do you think about the show and tell in the ART agenda? How do think your child experienced this?
5. What do you think about the chores (unpacking, packing and cleaning)? How do think your child experienced this?

Experiences

Here are a list of different skills your child might have acquired during the ART program.

- Do you think your child has now become better at some of these skills?
- Do you think that after participating in ART there are somethings that have become more difficult for your child?

  - Recognising emotions in face
  - Recognising emotions in body
  - Initiating a conversation
  - Managing conflicts
  - Managing stress
  - Joining a group
  - Understanding Social rules
  - Compromising
  - Saying No
  - Resolving conflicts
  - Going out to a café
  - Find new friends
  - Expressing emotions in your face
  - Expressing emotions in your body
  - Meeting new people
  - Social situations
  - Self confidence
  - Attending social event
  - Calling someone on the telephone
  - Taking turns
  - Listen to others
  - Offering or accepting help
  - Setting a meeting or date
  - feeling lonely
  - Understanding what others mean by what they are saying
  - How to get others to understand what you are saying

1. Which part of the program do you think has been most important for your child's social development?
2. In addition to the improvements in social skills, did participating in the ART program influenced your child in any other way?

3. Has participating in the ART program had any negative effects on your child? Has anything gotten worse as a result of their participation in the ART program?

4. During the time your child was participating in ART, how has your life been? Has there been any events that might have affected their behaviour during their participation in this program?

5. When you reflect on your child's participation in the ART program, what do your child's experience has been? Overall was participating in ART a positive or negative experience?

6. Is there anything you think the ART trainers should have done more of?

7. Is there anything you think the ART trainers should have done less of?

8. That is all on the questions, but please feel to share any information you think relevant concerning your child's participation in the ART program.

Thank you for being involved in our study!

Post ART Interview

Children

Hi, I Hope you have enjoyed attending the ART social program

I have not attended the ART program and I have no information about what happened in your group or what you, your group mates or trainers did during the ART sessions. That is why we are going through this interview so that you can let me know all about it.

STRUCTURE AND CONTENT

We are going to talk about some aspects of the ART program. This includes four questions. I am going to ask you to:

b. Tell me what happened
c. Say how you felt about them/experienced them
d. Say what was good about them
e. Say what was less good about them

Opening Round: At the beginning of each of the ART sessions you said your name and talked about the topic of day.

A. Can you tell me what happened (describe it)? (provide some examples)
B. On a scale of 1 to 10 how did you feel about it?

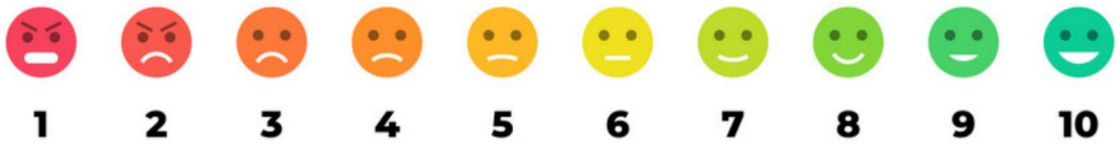

C. Can you tell me what was good about it?
D. Can you tell me what was less good about it?

How do you you feel?

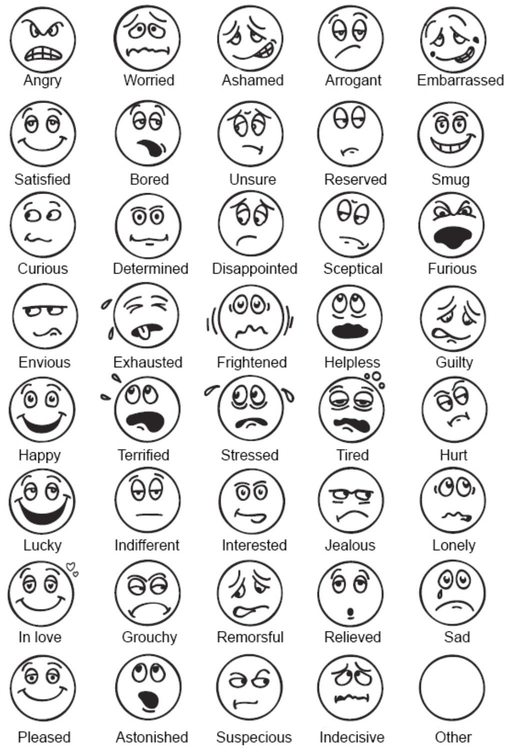

Emotion Thermometer

Describe how strong the feeling is!

10 = Very strong and 0 = Not strong at all

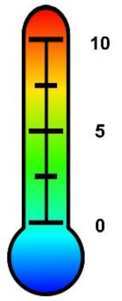

ART and Craft: Each session you prepared some materials and did some art projects.

A. Can you tell me what happened (describe it)? (provide some examples)
B. On a scale of 1 to 10 how did you feel about it?

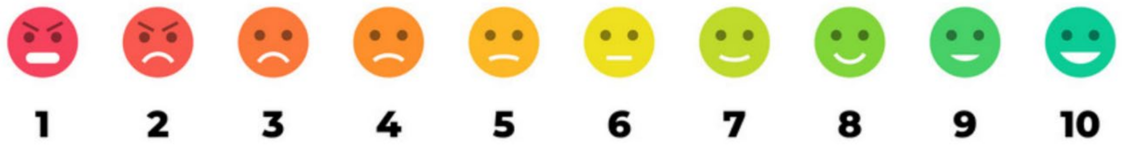

C. Can you tell me what was good about them?
D. Can you tell me what was less good about them?

Here is the list of activities covered in ART. <u>You might have only done some of them</u>.

- Superhero pop sticks
- Salt painting
- Music rain stick
- Cardboard maze
- Slime
- Treasure box
- Cloud dough
- The floor is lava/ make a path
- Hidden message
- My superpower rainbow
- Magic dragon/Magic wand
- Paper plate globe
- Dot painting
- Paper planes
- Origami
- Puzzles
- Parachute man
-

A. Which ones did you like? Why?

f. Which ones didn't you like? Why?

Snack Break: During these sessions there was a time when you could have a rest and eat something, right?

A. Can you tell me what happened (describe it)? (provide some examples)
B. On a scale of 1 to 10 how did you feel about it?

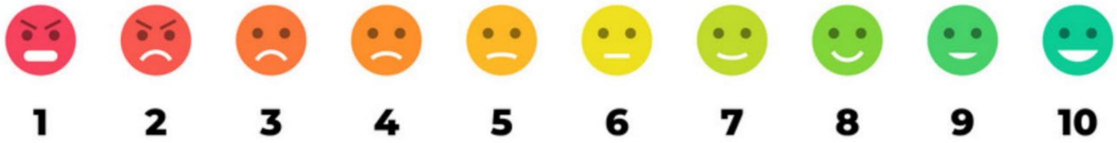

C. Can you tell me what was good about them?
D. Can you tell me what was less good about them?

Packing and Cleaning: Each session you packed away your ART work in the boxes. Can you tell me what happened (describe it)? (provide some examples)

A. On a scale of 1 to 10 how did you feel about it?

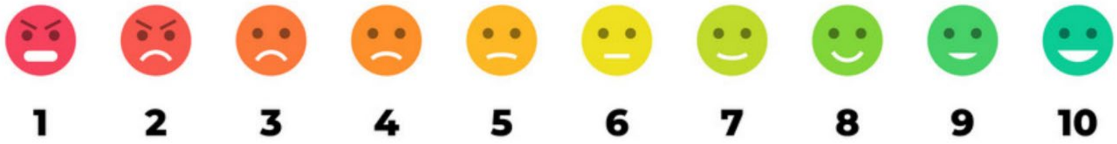

B. Can you tell me what was good about them?
C. Can you tell me what was less good about them?

Show & Tell: At the end of each session you had the chance to talk about your ART work, do you remember that?

A. Can you tell me what happened (describe it)? (provide some examples)
B. On a scale of 1 to 10 how did you feel about it?

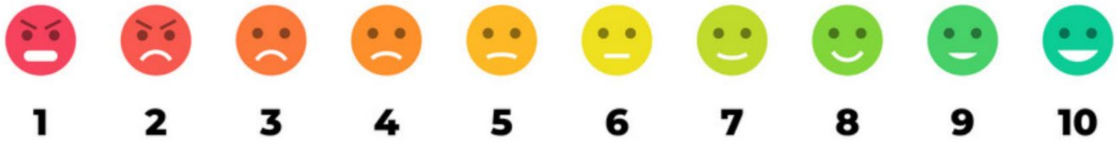

C. Can you tell me what was good about them?
D. Can you tell me what was less good about them?

Closing Round: At the end of each session there was a routine for ending or closing the session, do you remember that?

E. Can you tell me what happened (describe it)? (provide some examples)
F. On a scale of 1 to 10 how did you feel about it?

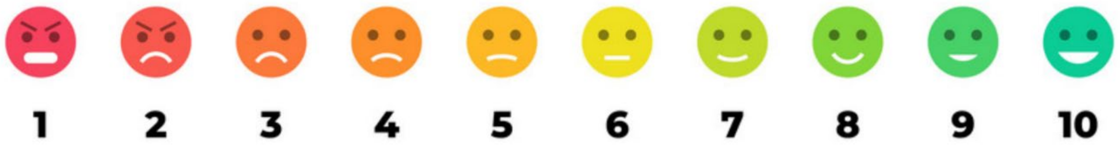

G. Can you tell me what was good about them?
H. Can you tell me what was less good about them?

Number of people in the group: What do you think about the number of people in your group? Was is there too many, or not enough?

A. Can you tell me about it (describe it)? (provide some examples)
B. On a scale of 1 to 10 how did you feel about it?

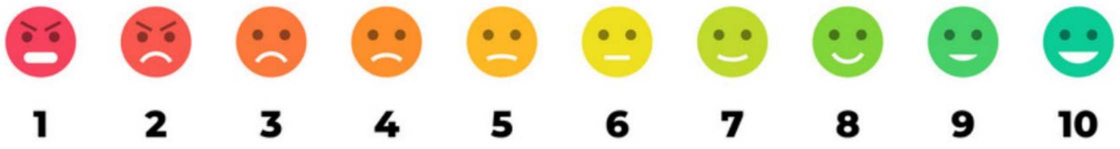

C. Can you tell me what was good about it?
D. Can you tell me what was less good about it?

Group Members: You had a few other people in the group. Did you like it? Did you make friends? (provide some examples)

A. Did you make friends? Can you tell me about it (describe it)? (provide some examples)
B. On a scale of 1 to 10 how did you feel about it?

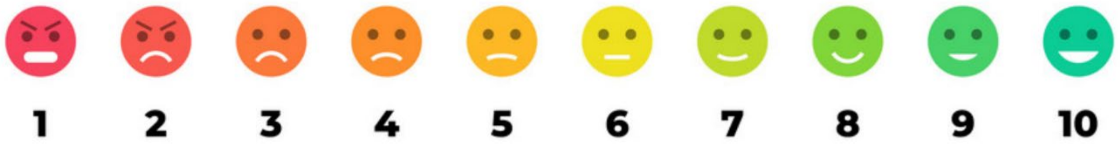

Experiences

Here is a list of skills different people have.

- Do you think by attending the ART group you have become better at some of these skills?

  Prompts: If so, which ones? Provide some examples.

- Do you think by attending the ART group, doing some of these skills have become more difficult for you?

  Prompts: If so, which ones? why do you think this is? Provide some examples.

- Recognising emotions in face
- Recognising emotions in body
- Initiating a conversation
- Managing conflicts
- Managing stress
- Joining a group
- Understanding Social rules
- Compromising
- Saying No
- Resolving conflicts
- Going out to a café
- Making new friends
- Expressing emotions on your face
- Expressing emotions with your body
- Meeting new people
- Social situations
- Self confidence
- Attending a social event
- Calling someone on the telephone
- Taking turns
- Listening to others
- Offering or accepting help
- Setting a meeting or date
- Feeling lonely
- Understanding what others mean by what they are saying
- How to get others to understand what you are saying

9. Did attending the ART program improve your social skills?

    Prompts: Can you tell me more about it? Why do you think this is?

10. Did attending the ART program impact your life in any other way? (For example, did you stop/start doing something else to attend the ART program?)

11. When you think back over the sixteen ART sessions, what do you think was your overall experience?

    A. Was attending ART program overall a positive or negative experience?
    B. Would you recommend ART groups to other teenagers with autism?

12. In the ART program is there anything you would have liked to have done more of?

13. In the ART program is there anything you would have liked to have done less of?

That is all of the questions I wanted to ask. Is there anything else you would like to share about your experience of attending the ART program?

Thank you for being involved in our study!
